# A prospective study of prognostic factors for duration of sick leave after endoscopic carpal tunnel release

**DOI:** 10.1186/1471-2474-10-144

**Published:** 2009-11-22

**Authors:** Torben Bæk Hansen, Jesper Dalsgaard, Anette Meldgaard, Kristian Larsen

**Affiliations:** 1 Department of Orthopaedics, Section of Hand Surgery, Regional Hospital Holstebro, Laegaardvej 12, DK-7500 Holstebro Denmark; 2 Orthopaedic Research Unit, Regional Hospital Holstebro, Laegaardvej 12, DK-7500, Holstebro Denmark; 3 Department of Orthopaedics, Section of Hand Surgery, Regional Hospital Herning, Gl. Landevej 61, DK 7400 Herning Denmark; 4 VIA University College, Gl. Struervej 1, DK-7500 Holstebro, Denmark; 5 Clinical Intitute, University of Århus, Brendstrupgårdsvej 100, DK-8200 Århus N, Denmark

## Abstract

**Background:**

Endoscopic carpal tunnel release with a single portal technique has been shown to reduce sick leave compared to open carpal tunnel release, claiming to be a less invasive procedure and reducing scar tenderness leading to a more rapid return to work, and the purpose of this study was to identify prognostic factors for prolonged sick leave after endoscopic carpal tunnel release in a group of employed Danish patients.

**Methods:**

The design was a prospective study including 75 employed patients with carpal tunnel syndrome operated with ECTR at two hospitals. The mean age was 46 years (SD 10.1), the male/female ratio was 0.42, and the mean preoperative duration of symptoms 10 months (range 6-12). Only 21 (28%) were unable to work preoperatively and mean sick leave was 4 weeks (range 1-4). At base-line and at the 3-month follow-up, a self-administered questionnaire was collected concerning physical, psychological, and social circumstances in relation to the hand problem. Data from a nerve conduction examination were collected at baseline and at the 3-month follow-up. Significant prognostic factors were identified through multiple logistic regression analysis.

**Results:**

After the operation, the mean functional score was reduced from 2.3 to 1.4 (SD 0.8) and the mean symptom score from 2.9 to 1.5 (SD 0.7). The mean sick leave from work after the operation was 19.8 days (SD 14.3). Eighteen patients (24%) had more than 21 days of sick leave. Two patients (3%) were still unable to work after 3 months. Significant prognostic factors in the multivariate analysis for more than 21 days of postoperative sick leave were preoperative sick leave, blaming oneself for the hand problem and a preoperative distal motor latency.

**Conclusion:**

Preoperative sick leave, blaming oneself for the hand problem, and a preoperative distal nerve conduction motor latency were prognostic factors for postoperative work absence of more than 21 days. Other factors may be important (clinical, demographic, economic, and workplace) in explaining the great variance in the results of sick leave after carpal tunnel release between studies from different countries.

## Background

Carpal tunnel syndrome (CTS) is a very common condition often treated surgically, and the outcomes of both open carpal tunnel release (OCTR) and endoscopic carpal tunnel release (ECTR) are generally excellent [1]. In spite of this, return to work may be prolonged, and some patients do not return to work at all after the operation. In a Norwegian study [2], 10% of the patients did not return to work after OCTR for CTS. Strenuous hand activity at work, being a female, and a workers compensation claim have been related to a predicted slower return to working activities [3,4]. Age and low preoperative hand function due to CTS have also been related to poor satisfaction after OCTR [3,5-8], and demographics, psycho-social factors, and work related factors may also be important for sick leave after surgical treatment of CTS [9].

Endoscopic carpal tunnel release with a single portal technique has been shown to reduce sick leave compared to OCTR as described by Agee [10], claiming to be a less invasive procedure and reducing scar tenderness leading to a more rapid return to work. We have used this technique since 1995 as a standard procedure in CTS, and the purpose of this study was to identify prognostic factors for prolonged sick leave after ECTR in a group of employed Danish patients.

## Methods

The design was a prospective study of employed patients operated with ECTR at two hospitals. The two hospitals (Regional Hospital Holstebro and Regional Hospital Herning) are part of the same hospital organisation (Hospital Unit West), and the Section of Hand Surgery covers both hospitals. The operative procedures are the same, and in both hospitals ECTR is the standard method for carpal tunnel release. Only patients with a history of wrist fracture, rheumatic disease or former carpal tunnel release are treated with OCTR. All patients who were employed and having ECTR were prospectively included at both hospitals. The sample size was set to 75 employed patients based on sick leave data from a pilot study of the same sample size with a mean sick leave of 3 weeks. The Microaire single portal technique [10] was used in all patients as an out-patient procedure under intravenous analgesia or local anaesthesia. Only patients undergoing unilateral operation were included. At base-line before the operation and at the 3-month follow-up visit at hospital, a self-administered questionnaire was collected concerning job demands on hand function, physical, psychological, and social circumstances in relation to the hand problem: 1. Do you find your job to be hand demanding? 2. Do you think that you would be able to use your hand normally 3 months after the operation? 3. Are you afraid of getting chronically problems with your hand? 4. Do you blame yourself for the hand problem? 5. What do you think about your general health status? 6. How supportive are your family and friends? 7. Do you feel yourself alone with your hand problems? 8. If you look at your work, salary, carrier possibilities, management and colleagues as a whole how satisfied are you? All questions were to be answered on a rank ordinal scale from 0-10, where 0 was "Not at all" and 10 was "Very much". At both visits the data set were collected and controlled for missing data. The questions regarding symptoms and function were based on symptom and functional scores for persons with CTS adapted from the Boston questionnaire [11] and translated into Danish. Data from a nerve conduction examination were collected at baseline and at the 3-month follow-up, and only patients with abnormal preoperative nerve conduction values were included in the study. Positive changes in nerve conduction were defined as the absolute change from the preoperative conduction value to the postoperative value ie. an improvement in the measured values except for distal motor latency where improvement would be a reduction in the measured value. The duration of sick leave was self-reported by the patients, and duration of sick leave was not specified from the hospital but was determined by the general practitioners.

### Statistics

Primary outcomes were cumulated days off work after ECTR. Off work was dichotomised at 21 days or below against above 21 days based on the results from the pilot study. Secondary outcomes were analysed by both univariate logistic regression and multivariate logistic regression. Candidate variables for multivariate analysis were variables with a significant result from the univariate analysis. In the multivariate analysis, significant candidates were used in the models two by two and with all variables together. The significant level was set at *P *< 0.05 and all confidence intervals (CI) represent 95% CI. Data were entered by using EpiData and 15% of the data were entered twice. After initial data entering showed no data entry error, the rest of the data was entered only once. Analysis was performed by using SPSS package version 11.0 for Windows.

The study was approved by The Ethical Comite of Ringkjøbing, Ribe and Sønderjyllands amter (ref. nr. 2505-03) and informed consent has been retrieved from all patients.

## Results

**Figure 1:**
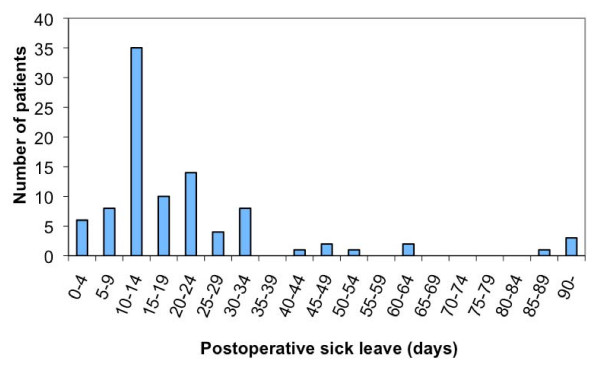
**Sick leave after the operation**.

In all, 75 employed persons operated on 75 hands were included in the study during a 10-month period, and none of the patients were lost at follow-up. In the same period 26 non-employed patients were treated with ECTR. The mean age was 46 years (SD 10.1), the male/female ratio was 0.42, and the mean preoperative duration of symptoms 10 months (range 6-12). Only 21 (28%) were unable to work preoperatively, and these had a mean sick leave of 4 weeks (range 1-4). Twenty-eight (37%) patients reported their job to be demanding on hand function (score>7). The mean functional score, the mean symptom score and ENG recordings improved after the operation (Table 1). There was a correlation between improvement in function and improvement in distal motor latency (table 2), but otherwise no correlation was found between changes in nerve conduction and change in function or between changes in nerve conduction and change in symptoms (Table 2, 3). The mean sick leave from work after the operation was 19.8 days (SD 14.3) (Figure 1.). Forty-nine patients (65%) returned to work during the first 14 days. Eighteen patients (24%) had more than 21 days off work. Two patients (3%) were still unable to work after 3 months due to hand problems. Both patients were unable to work before the operation. None of the patients received postoperative physiotherapy, and there were no infections, cases of nerve damage or RSD. In the univariate regression analysis statistically significant prognostic findings for more than 21 postoperative sick days were preoperative sick leave, severity of preoperative symptoms, a preoperative ENG finding of a distal motor latency, thoughts of change of work, and blaming oneself for the hand problem (Table 4). On multiple logistic regressions, severity of preoperative symptoms and thoughts of change of work were not significant prognostic factors and were excluded. All the remaining three factors were still a significant prognostic factor for sick leave of more than 21 days (table 5).

**Table 1:** Changes in function, symptoms and ENG 3 months after the operation

	**Preoperative (mean)**	**3 months postoperative (mean)**
Function (Boston score)	2,3 (SD 0,8)	1,4 (SD 0,6)
Symptoms (Boston score)	2,9 (SD 0,7)	1,5 (SD 0,5)
ENG: distal motor latency (ms)	5.2 (SD 1,3)	4,5 (SD 1,1)
ENG: sensory response finger 2 (ms)	40.0 (SD 7.2)	45,9 (SD 6,0)
ENG: amplitude finger 2 (μV)	10.4 (SD 7.2)	12,3 (SD 7,0)
ENG: sensory response finger 3 (ms)	39.0 (SD 6.4)	44,8 (SD 6,3)
ENG: amplitude finger 3 (μV)	9.0 (SD 7.0)	11,5 (SD 6,7)
ENG: conduction from palm to wrist (ms)	35.5 (SD 7.0)	39,8 (SD 6,7)

**Table 2:** Changes in nerve conduction versus change in function analyzed with linear regression test.

	**Regression** **Coefficient**	**95% C.L**.
distal motor latency (ms)	0,300*	0,057 - 0,537
sensory response finger 2 (ms)	0,018	-0,019 - 0,054
amplitude finger 2 (μV)	0,039	-0,001 - 0,080
sensory response finger 3 (ms)	0,008	-0,030 - 0,045
amplitude finger 3 (μV)	0,031	-0,007 - 0,068
conduction from palm to wrist (ms)	0,012	-0,027 - 0,050

* P < 0,05

**Table 3:** Changes in nerve conduction versus change in function analyzed with linear regression test.

	**Regression** **Coefficients**	**95% C.L**.
distal motor latency (ms)	0,012	-0,156 - 0,181
sensory response finger 2 (ms)	-0,007	-0,036 - 0,023
amplitude finger 2 (μV)	0,006	-0,027 - 0,039
sensory response finger 3 (ms)	-0,014	-0,044 - 0,015
amplitude finger 3 (μV)	0,006	-0,025 - 0,037
conduction from palm to wrist (ms)	-0,008	-0,038 - 0,023

*P < 0,05

**Table 4:** Univariate logistic regression of prognostic factors for postoperative sick leave of more than 21 days after ECTR in 75 hands in 75 patients

**Prognostic factors**			**OR 21 days**	**95% CI**
Known risk factors				
High demands on hand function at work	> 7 (scale 0-10)	28 (37%)	1.28	0.96 - 1.71
Gender: Male:Female	Female	53 (71%)	0.62	0.18 - 2.15
Workers compensation case: yes/no	Yes	5 (7%)	1.47	0.15 - 14.0
Preoperative sick leave: yes/no	Yes	21 (28%)	9.43*	2.84 - 31.3
				
Physical factors				
Age	Years	Mean 46 (SD 10.1)	1.00	0.95 - 1.06
Duration of hand problems	Months	10 (6;12)**	1.30	0.95 - 1.77
Preoperative sick leave	Weeks	4 (0;15)**	1.04	0.99 - 1.10
Complicating diseases yes/no	Yes	8 (11%)	1.69	0.36 - 7.88
Self-reported health status	<3 (scale 0-10)	2 (0;5)	1.07	0.84 - 1.38
Preoperative functions (Boston questionnaire)	Score>2***	2,3 (SD 0,8)	1.80	0.81 - 4.02
Preoperative symptoms (Boston questionnaire)	Score>2***	2,9 (SD 0,7)	3.51*	1.32 - 9.37
ENG: Preoperative distal motor latency	Ms	5.2 (SD 1,3)	1.37*	1.01- 1.87
ENG: Preoperative sensory response finger 2	Ms	40.0 (SD 7.2)	0.96	0.89 - 1.03
ENG: Preoperative amplitude finger 2	μV	10.4 (SD 7.2)	0.99	0.91 - 1.07
ENG: Preoperative sensory response finger 3	Ms	39.0 (SD 6.4)	0.95	0.87 - 1.04
ENG: Preoperative amplitude finger 3	μV	9.0 (SD 7.0)	0.98	0.90 - 1.07
ENG: Preoperative conduction from palm to wrist	Ms	35.5 (SD 7.0)	0.94	0.86 - 1.03
				
Psychological factors				
Afraid of having chronic hand problems	>7 (scale 0-10)	5 (0;10)**	1.08	0.94 - 1.24
Blaming oneself for the hand problem	>7 (scale 0-10)	2 (0;6)**	1.25*	1.03 - 1.52
Feeling of being alone with hand problems	>7 (scale 0-10)	2 (0;8)**	1.04	0.87 - 1.23
Belief in cure of hand problems 3 months after the operation	>7 (scale 0-10)	1 (0;5)**	1.22	0.94 - 1.57
				
Social factors				
Consideration of job change because of hand problems yes/no	Yes	15 (20%)	4.68*	1.39 - 15.7
Support from family and friends	<3 (scale 0-10)	2 (0;7)**	0.93	0.75 - 1.15
Job satisfaction	<3 (scale 0-10)	2 (0;6)	1.09	0.88 - 1.37

* *P *< 0.05

** medians with 10% and 90% percentiles

***dichotomized based on clinical experience.

**Table 5:** Multivariate logistic regression analysis of prognostic factors for more than 21 days of sick leave based on two by two variable analysis and all variables analysis

	**All**	
**Prognostic factors**	**OR**	**95%C.L**.
Preoperative sick leave	7.40*	2.12-25.03
Preoperative distal motor latency	1.74*	1.14-2.41
Blaming oneself for the hand problem	1.26*	1.01-1.52

*p < 0.05

## Discussion

In a leading article in the BMJ, Graham [12] concluded that the outcomes of carpal tunnel release are generally excellent, and surgery - whether open or closed - works if the diagnosis is right, but outcomes are generally less favourable in elderly people, although patient satisfaction seems to be the same as in younger patients. This observation is based on studies indicating that age may be an important factor as a predictor of prognosis in the surgical treatment of carpal tunnel syndrome [3,5-8]. We did not find any correlation between age and gender and return to work after 21 days, but age may not be a good predictor of return to work. Studies indicating age as a predictor of less favourable outcome after carpal tunnel show that results are less favourable after 65-70 years of age, at which time most patients are retired. This is supported by a large retrospective study by Katz [13], in which age was not a strong predictor of outcome. The strongest predictors of a less favourable outcome of surgery in this study were worse scores on patient-reported measures of upper extremity functional limitation and mental health status, alcohol abuse, and the involvement of an attorney. Symptom and functional scores did not show any significant effect on postoperative sick leave in our study as they did in studies by Carmone and Atroshi [3,7]. The Boston questionnaire has not been validated into Danish and we used non-validated cut points in the analysis based on clinical experience alone, and this may have flawed our analysis. On the other hand, preoperative sick leave was a very strong prognostic factor for postoperative sick leave in our study as in other studies [4], but there does not seem to be an association between the length of the preoperative sick leave and the length of the postoperative sick leave. Using sick leave as a measure of outcome is in it self also a problem, as this may not be the same as the disability to work. In Denmark all patients have full salary on sick leave so different reimbursement possibilities do not influence sick leave in our study. We have tried to identify psychological and social factors and include these factors in the analysis, but many other factors may influence sick leave after the operation. The surgeons or general practitioners advice may be an important factor. In our study we tried not to bias the study by giving the patients specific advice about the postoperative sick leave, but were not able to avoid the general practitioners advising the patients specifically about sick leave. Other factors may also influence postoperative sick leave as experience reported from friends and relatives about sick leave after a hand operation. Workers' compensation claims are known to be a negative factor in prognosis [3,4,9,14], but also here, we found no correlation. In our pilot study "White collar" and "Blue collar" was not a predictor of return to work. This distinction between workers may not be relevant anymore, as job functions has changed both for "Blue collar" and "White collar" workers. Industrial workers may now be placed in functions that are not very hand demanding and "White collar" workers may have very hand demanding tasks using computers. As a result we have used the patients self reported evaluation of hand demands in their job instead of "White collar" and "Blue collar". Other studies have shown a positive correlation between hand activities at work and sick leave [3,7], but we did not find any correlation between high demand on hand function at work and return to work after 21 days.

ECTR is a minimally invasive technique that in some studies has reduced sick leave compared with OCTR [1]. It may also facilitate postoperative rehabilitation during the first weeks after the operation because of less pain [15], but at a significant extra cost to the hospital [16]. We used ECTR in our study, and in a recent Cochrane review, Sholten [1] concluded that none of the existing alternatives to standard open carpal tunnel release offered significantly better relief from symptoms in the short- or long-term, but some evidence indicates that ECTR may reduce time off work. Mean sick leave in our study was only 20 days, and only 3% were still on sick leave after 3 months. Katz [9] found 29% on sick leave after 2 months and 19% still on sick leave after 6 months. Comparing OCTR with ECTR, Saw [16] found a mean sick leave of 18 and 26 days, respectively. Atroshi [15] found a mean of 28 days in both groups comparing OCTR with ECTR. In a study from Norway by Bekkelund [2], the mean sick leave was 7 weeks after OCTR. This indicates that the type of surgery is probably not the only important factor determining postoperative sick leave.

The preoperative electrodiagnostic test is considered the gold standard for diagnosis in carpal tunnel syndrome [17,18], and preoperative nerve conduction values may be a good predictor of outcome [8]. Indeed, in some studies, improvement in nerve conduction has been a better predictor of outcome than the Boston questionnaire [19,20], although not in all studies. In a Swedish study [7], no correlation was found between nerve conduction and postoperative sick leave in patients after ECTR, and other studies [21] have shown the same to be true after OCTR. In our study, a preoperative distal motor latency was the only significant prognostic factor for duration of postoperative sick leave, indicating that only severe nerve conduction abnormalities with distal motor latency (often paired with atrophy of the thenar) result in impaired hand function and prolonged and sometimes incomplete rehabilitation.

In our study, we found psychosocial factors such as blaming yourself and consideration of a job change to be prognostic factors for sick leave. We used a non-validated questionnaire to identify these factors and non-validated cut points in the analysis. This may have flawed the analysis but the results indicate that psychosocial factors play an important role in sick leave after ECTR, and many other factors than symptoms, surgical technique, hand function, and improvement in nerve conduction may be important for determining duration of sick leave after the operation.

## Conclusion

In this Danish study, most patients rapidly returned to work after endoscopic carpal tunnel release, and the most important predictors of absence from work of more than 21 days after carpal tunnel release were preoperative sick leave, blaming oneself for the hand problem and a preoperative distal nerve conduction motor latency. Other factors may be important (clinical, demographic, economic, and workplace) in explaining the great deviations in the durations of sick leave after carpal tunnel release reported in studies from different countries.

## Competing interests

The authors declare that they have no competing interests.

## Authors' contributions

TBH did the data collection and interpretation of the results, did the manuscript preparation, and have read and approved the final manuscript. JD also did the data collection and interpretation of the results, and have read and approved the final manuscript. AM did the statistical analysis and interpretation and have read and approved the final manuscript. KL did the statistical analysis and interpretation and have read and approved the final manuscript.

## Pre-publication history

The pre-publication history for this paper can be accessed here:

http://www.biomedcentral.com/1471-2474/10/144/prepub
